# Efficacy of astragalus in the treatment of radiation-induced lung injury based on traditional Chinese medicine: A systematic review and meta-analysis of 25 RCTs

**DOI:** 10.1097/MD.0000000000030478

**Published:** 2022-09-09

**Authors:** Xue-Meng Pang, Hou-Hao Cai, Jie Zhao, Ping-Yi Sun, Jing-Jing Shi, Yan-Li Zhang, Juan Liu, Zong-Chen Liu, Xin Zheng

**Affiliations:** a Shandong University of Traditional Chinese Medicine, Jinan, China; b Jinan Zhangqiu District Traditional Chinese Medicine Hospital, Jinan, China; c Qingdao Hospital of Traditional Chinese Medicine (Qingdao Hiser hospital), Qingdao, China.

**Keywords:** astragalus, meta-analysis, radiation pneumonitis, radiation pulmonary fibrosis, radiation-induced lung injury, systematic review

## Abstract

Astragalus (Hedysarum Multijugum Maxim., Huangqi) is a Chinese herbal medicine, and according to the theory of traditional Chinese medicine (TCM), Chinese medicinal preparations containing astragalus can be used clinically to treat radiation-induced lung injury (RILI). To systematically review the efficacy and safety of Chinese medicinal preparations containing astragalus in the prevention and treatment of RILI by means of meta-analysis.

**Methods:**

A systematic literature on randomized controlled trials (RCTs) of prescriptions containing astragalus in the treatment of RILI by Pubmed, Embase, Web of Science, Cochrane Library, China Biomedical Literature Database, China National Knowledge Infrastructure, China Science and Technology Journal Database, WANFANG Database. The retrieval time is from the establishment of the database to January 18, 2022. Meta-analysis, heterogeneity test and sensitivity analysis were performed on eligible RCTs using Revman 5.4 software and STATA 17.0 software, and a “funnel plot” was used to analyze potential publication bias.

**Results:**

A total of 25 RCTs were included, including 1762 patients, and the most widely used drugs were heat-clearing and detoxifying, yin-nourishing and qi-nourishing. The prescriptions containing astragalus can significantly reduce the total incidence of RILI (*P* < .01), improve the total effective rate and cure rate of RILI (*P* < .01), improve the quality of life of patients, alleviate breathing difficulties and reduce the expression of inflammatory factors (*P* < .01), and no adverse reactions related to TCM treatment were reported.

**Conclusion:**

The traditional Chinese medicinal preparation containing astragalus can effectively alleviate the clinical symptoms of RILI, reduce the toxic side effects, and is safe to use in clinic.

## 1. Introduction

Targeted radionuclides using microspheres containing radioactive substances and other monoclonal antibody systems labeled with radioactive substances are currently common treatment methods for the treatment of malignant tumors of the chest.^[1]^ Radiotherapy (RT) includes intensity modulated RT, image guided radiotherap, and stereotactic RT, external beam RT.^[2]^ Common effects of thoracic RT include radiation-induced lung injury (RILI), radiation-induced heart disease, and anxiety, depression, fatigue and so on.^[3]^ RILI includes any pulmonary toxicity caused by RT, which is acutely manifested as radiation pneumonitis (RP) and long-term manifestation as radiation pulmonary fibrosis (RPF).^[1]^ For very mild symptoms, clinical observation can be considered. Most experts recommend systemic glucocorticoids to treat significantly symptomatic RP.^[1,4]^

However, the treatment of RPF is mainly to relieve symptoms, and there is a lack of effective treatment measures. At this time, Chinese medicine and traditional Chinese medicine (TCM) play a major advantage, and the research technology and means for natural medicine are also constantly improving. Bioassays of genotoxicity and mutagenicity of compounds of Eleutherine plicata Herb using modern techniques, computational assessment of the potential toxicity of these molecules using PreADMET server, molecular docking and molecular dynamics to gain insight into the genotoxicity and mutagenic potential of isoeleutherin and eleutherin.^[5]^ Using chemometrics, multivariate analysis and molecular docking, the target properties of drugs in biological systems are calculated and correlated with antimalarial activity, resulting in the analysis of the most promising compounds for the treatment of malaria.^[6]^ Ligand-based and target-based potential inhibitors were identified using virtual screening, pharmacophore modeling and molecular docking research methods from the ZINC database to obtain anti-tuberculosis natural source compounds with dual-action drugs that inhibit the same biological target.^[7]^ In traditional medicine, the bioactive compounds of essential oils extracted from spice plants such as Cinnamomum zeylanicum, Mentha piperita, Ocimum basilicum, Origanum vulgare, and Rosmarinus officinalis have antioxidant activity and scavenge free radicals.^[8]^ The Amazonian flora has a wide range of aromatic plants and is used in traditional medicine to treat a variety of diseases, while the bioactive compounds in the essential oils of the Amazonian biome in natural medicine are resistant to A549 cells and NCI-H460 lung cancer cells.^[9]^ Determination of the chemical composition of essential oils of I. asarifolia and I. setifera using gas chromatography combined with gas-chromatography-mass-spectrometry and gas chromatograph equipped with a flame ionization detector and machine learning and other in silico associated major toxicity, showing permeability to the blood-brain barrier and gastrointestinal tract.^[10]^

In TCM, RILI is divided into incubation period, acute period, and advanced period. They are respectively given to regulate qi and nourish yin, moisten lung and relieve cough; clear heat and detoxify, release lung qi, and resolve phlegm.^[11]^ The study by Xia et al showed that astragalus membranaceus can reduce the deterioration of pulmonary diffusion function after RT, have protective effects on early RP and advanced pulmonary fibrosis, and long-term application has no damage to heart, liver, and kidney functions.^[12]^ Tian et al analyzed the prescription rules of Chinese herbal medicines for the treatment of RILI through the complex network of Chinese medicines and found that the main ones were Ophiopogon japonicus and Astragalus.^[13]^ Shenqi Fuzheng Injection (SFI) was composed of Codonopsis pilosula and Astragalus extracts. Dong et al determined the protective effect of SFI on RILI with C57BL/6 single-dose irradiation, and found that tumor necrosis factor (TNF)-α and transforming growth factor (TGF)-β were the key mediators in the pathogenesis of RILI, and SFI decreased expression of TNF-α and TGF-β1 after RT.^[14]^ Cryptotanshinone, a major lipophilic extract from Salvia miltiorrhiza Bunge (Danshen), protects lung function and alleviates RILI in rats, presumably by inhibiting CCL3/CCR1 activation to modulate inflammatory and fibrotic cytokines realized.^[15]^ Yangyin Qingfei Decoction has a protective effect on RILI in rats, possibly by down-regulating the expression of MMP-12 and TIMP-1, which leads to the balance of extracellular matrix synthesis and degradation, and alleviates the occurrence of pulmonary fibrosis.^[16]^ Intervention of BABL/C mice with flavonoids extracted from astragalus after exposure to 10 Gy thoracic radiation showed up-regulation of mouse SOD levels, while down-regulation of TGF-β1 and IL-6. The expression of TGF-β1 and TNF-α was significantly increased in alveolar epithelial cells and alveolar septal macrophages, suggesting that flavonoids may act as protective agents against RILI.^[17]^

In this paper, a systematic review and meta-analysis of traditional Chinese medicinal preparations containing astragalus in the treatment of RILI were conducted in order to provide a good prospect for the clinical treatment of RILI.

## 2. Data and Methods

### 2.1. Protocol and registration

This systematic review and meta-analysis was registered in PROSPERO, an international prospective register of systematic reviews, with the registration number CRD42022297067 (available from https://www.crd.york.ac.uk/prospero/).

### 2.2. Search strategy

The search strategy and inclusion criteria were decided according to the guidance of the PRISMA agreement.^[18]^ Computer searched Pubmed, Embase, Web of Science, Cochrane Library, China Biomedical Literature Database, China National Knowledge Infrastructure, China Science and Technology Journal Database, WANFANG Database. The retrieval time is from the establishment of the database to January 18, 2022. Retrieval adopts the combined search of subject headings and free words. The search terms were (“radiation pneumonitis” OR “radiation lung injury” OR “radiation pulmonary fibrosis” OR “radiation lung disease”) AND (“traditional Chinese medicine” OR “Chinese herbal medicine” OR “Hedysarum Multijugum Maxim” OR “astragalus” OR “Astragalus propinquus” OR “huangqi” OR “radix astragali” OR “Milk Vetch*”) AND (“randomized controlled trial” OR “random”). Taking pubmed as an example, the retrieval formula is (((random) OR (randomized controlled trial[MeSH Terms])) AND ((astragalus plant*[MeSH Terms]) OR ((((((((((astragali) OR (astragalus membranaceus)) OR (astragalus)) OR (astragalus root)) OR (milkvetch root)) OR (radix astragali)) OR (astragalus propinquus)) OR (astragali radix)) OR (Wooly Locoweed*)) OR (Milk Vetch)))) AND ((radiation pneumonitis[MeSH Terms]) OR (((((((((((((((((radiation pneumonia*) OR (radiation-induced lung injury)) OR (adiation induced pulmonary fibrosis)) OR (radiation-induced lung damage)) OR (Radiation Pulmonary Fibrosis)) OR (adiation induced lung fibrosis)) OR (radiation fibrosis)) OR (radiation lung disease)) OR (radiation-induced injuries)) OR (irradiation damage, pneumonia)) OR (irradiation injury, pneumonia)) OR (irradiation pneumonia)) OR (pneumonia radiationis)) OR (radiation trauma, pneumonia)) OR (radiation injury, pneumonia)) OR (radiation damage, pneumonia)) OR (radiation damage, pneumonia))). Two authors (X-MP and Y-LZ) independently conducted manual searches in the above-mentioned English and Chinese electronic databases, the language is limited to both Chinese and English and preliminarily obtained the corresponding literature, which was retrieved until January 2022. Our literature search was limited to human randomized controlled trials (RCTs), searched original references and manually reviewed articles for potentially relevant trials, and attempted to identify grey literature through other sources.

### 2.3. Inclusion and exclusion criteria

#### 2.3.1. Inclusion criteria.

The subjects of the study were patients with thoracic malignancy diagnosed by pathology or cytology (over 18 years old, no upper limit of age), who received chest RT, and the irradiation dose was not limited; Interventions in the control group: on the basis of RT, they received non-specific treatments such as placebo, symptom support, adrenal corticosteroids, and broad-spectrum antibiotics; interventions in the treatment group: on the basis of the control group, TCM preparations containing astragalus were added, and the dosage form and mode of administration are not limited; The research type is public or unpublished RCTs with full text available; The outcome indicator is one of the following auxiliary outcome indicators included in the literature: Recent Response Evaluation; Acute radiation injury; Cancer patient quality of life score; Cytokine levels; TCM clinical symptoms improvement or integral change.

#### 2.3.2. Exclusion criteria.

Full-text conference abstracts or scientific and technological achievements cannot be obtained; Animal experiments, review, case cases, systematic evaluation, conference papers, non-RCTs; Duplicate publications; The enrolled patients received RT to other parts of the chest at the same time, or received chest RT within 1 year before treatment; Study subjects combined with other lung diseases; Self-made preparations with unknown ingredients.

### 2.4. Data screening and extraction

Two researchers (J-JS and JZ) independently searched the above electronic databases according to the inclusion and exclusion criteria and extracted the following data from each article: author name, age, gender, publication date, sample size, trial group and the intervention measures of the control group, the formulation and composition of traditional Chinese medicinal preparation containing astragalus, the route of administration, the remission rate and adverse drug reactions, the quality of life, the immune function, the lung function and the level of cellular inflammatory factors. Two-person interactive screening was used to extract literature, and disputes were resolved through a third party (P-YS), or the authors were contacted when necessary.

### 2.5. Quality assessment

The assessment followed the Cochrane Handbook for Systematic Reviews of Interventions,^[19]^ including whether the method of random assignment was used; whether the results of random assignment were strictly implemented; whether blinding was used; whether the outcome data were complete; whether the study results were selectively reported; whether there are other sources of bias. The quality assessment was done independently by 2 authors (JL and Z-CL). There was disagreement on risk of bias in specific studies, and disputes were resolved through a third party (H-HC), or the authors were contacted when necessary.

### 2.6. Statistical analysis

This systematic review and meta-analysis was performed by RevMan 5.4 software for literature quality assessment and Stata 17.0 for statistical analysis. For qualitative data, the relative risk (RR) and 95% confidence interval were used. For quantitative data, weighted mean difference was used as the effect analysis statistic. *Q* test and *I*^2^ test were used to determine whether there was heterogeneity between studies. If the risk of heterogeneity is small (*I*^2^ ≤ 50%), the fixed effect model will be used for combined analysis; if the risk of heterogeneity among the results of each study is large but within the acceptable range (*I*^2^ > 50%), and the combined effect size of the random effect model cannot be found, subgroup analysis, sensitivity analysis and regression analysis are used to try to find out the reasons for heterogeneity, or descriptive analysis is performed; if only statistical heterogeneity is used, random effect model is used for combined analysis. When the number of studies was sufficiently large, a funnel plot test was performed to observe whether there was publication bias.

## 3. Results

### 3.1. Results of literature search

**Figure 1.**
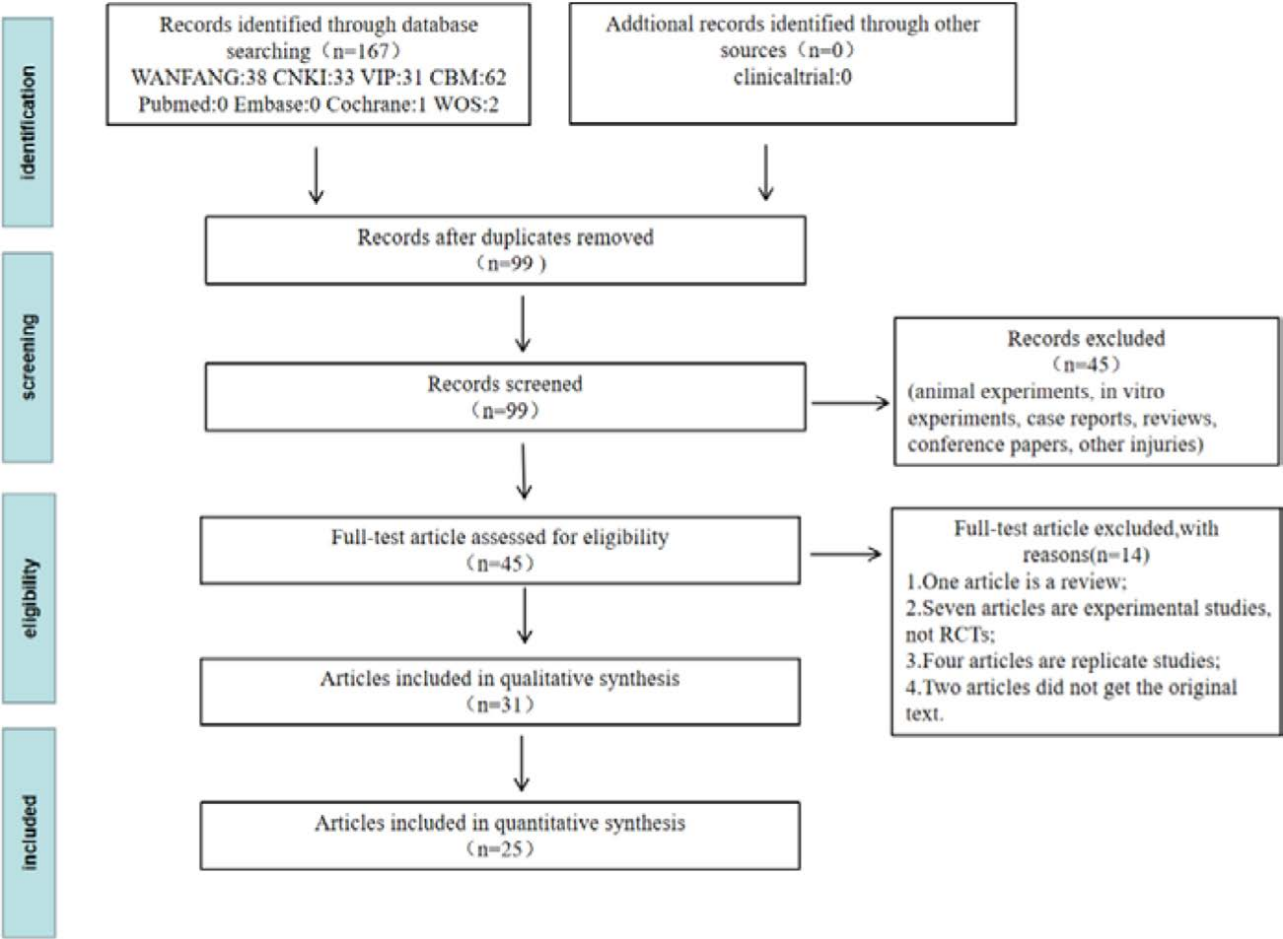
Flow chart of the literature search in the PRISMA format. Abbreviations: CBM = China biomedical literature database, CNKI = China national knowledge infrastructure, RCT = randomized controlled trial, WOS = web of science; VIP = China science and technology journal database.

First, 167 potentially eligible articles were retrieved. Forty-five literatures were initially obtained by reading the title and abstract of each article. Animal experiments, in vitro experiments, case reports, reviews, conference papers, other site injuries and other articles that did not meet the requirements of the inclusion criteria were excluded. After obtaining the full text of these 45 literatures and browsing, excluding the non-compliant interventions, not RCTs, not lung injury, and duplicate literatures, 25 literatures were finally included. Flowchart Results of the literature screening and study selection process are shown in Figure 1 and Table 1.

**Table 1:** Detailed characteristics of included trials.

First author (year)	Sample size (T/C)	Mean ge (year) (T/C)	Gender (M/F)	Type of RT dose of RT	Tumor or histological type	TNM stage	Intervention group	Route of administration	Evaluation indicators
Zhao^[20]^	20/20	58.15 ± 10.56	27/13	3D-CRT	lung cancer	-	Aidi injection	intravenous drip	①②③④
(2013)		56.85 ± 11.03		DT50–66 Gy					
				(2 Gy/f)					
Sun^[21]^	86/45	≥65	82/49	3D-CRT	NSCLC	IIb,	Shenqifuzheng injection	intravenous drip	①③
(2014)				DT60–70 Gy		III,			
				(2 Gy/f)		IV			
Liu^[22]^	26/32	65 (20~81)	41/17	3D-CRT	lung cancer,	IIIa,	Shenqifuzheng injection	intravenous drip	②④
(2007)		63 (17~86)		DT50.4–70.2 Gy	esophageal cancer,	IIIb			
					mediastinal lymphoma				
Ba^[23]^	52/64	-	-	3D-CRT	NSCLC,	IIIa,	Shenqifuzheng injection	intravenous drip	④
(2016)				DT63–70.2 Gy	esophageal cancer	IIIb			
Hu^[24]^	20/20	Total	24/16	3D-CRT	NSCLC	IIb,	Shenqifuzheng injection	intravenous drip	①⑤
(2013)		(60.2 ± 10.6)		DT70 Gy		IIIa,			
				(2 Gy/f)		IIIb,			
						IV			
Wang^[25]^	21/21	Total	-	3D-CRT	NSCLC	IIIa,	Shenqifuzheng injection	intravenous drip	①⑤
(2009)		72 (70~85)		DT70 Gy		IIIb,			
				(2 Gy/f)		IV			
Tan^[26]^	50/50	57.60 ± 7.04	55/45	RT	adenocarcinoma,squamous cell carcinoma,others	I,	Shenqigujin Tang	per os	①④
(2020)		58.22 ± 7.30				II,			
						III,			
						IV			
Gao^[27]^	79/79	70~81	87/71	3D-CRT	NSCLC	I,	Shenqishiyiwei Granules	per os	①③
(2012)		67~85		DT60–66 Gy		II,			
				(1.8 Gy/f)		III,			
						IV			
Wang^[28]^	33/31	60.91 (43~77)	35/29	CRT	NSCLC	III,	Shenqiyifei Syrup	per os	①
(2017)		59.1 (44~76)		DT60–66 Gy		IVa,			
				(2 Gy/f)		IVb			
Ran^[29]^	29/29	66.2 (61~70)	38/21	RT	lung cancer	IIb,	Shenqiyifei Syrup	per os	②
(2012)		65.7 (60~71)		DT68–70 Gy		IIIa,			
				(2 Gy/f)		IIIb			
Zou^[30]^	32/30	48.4 ± 3.9	44/18	CRT	lung cancer	IIa,	Shenqiyifei Syrup	per os	①②
(2015)		50.4 ± 4.1		DT70 Gy		IIb,			
				(2 Gy/f)		IIIa,			
						IIIb			
Xu^[31]^	30/30	66.95 ± 9.42	33/27	RT	-	-	Danqimaidi Tang	per os	①
(2014)		67.53 ± 4.12							
Shen^[32]^	30/30	50 ± 10	25/35	CRT + 3D-CRT	breast tumor	-	Huangqijing Oral Liquid	per os	②
(2009)		50 ± 10		DT30–75 Gy					
Zhang^[33]^	30/31	-	48/13	3D-CRT/IMRT	lung cancer	I,	Huangqifuzheng Granule	per os	④
(2015)				DT60–66 Gy	esophageal cancer	II,			
				(2 Gy/f)		III,			
Liu^[34]^	16/10	-	16/10	CRT	lung cancer,	-	Huangqi Injection	intravenous drip	②
(2005)				DT39–64 Gy	esophageal cancer,				
					mediastinal lymphoma,				
					breast cancer				
Ma^[35]^	30/30	54.3 ± 10.97	42/18	CRT	lung cancer,	-	Huangqisanshen Yin	per os	②③
(2014)		54.37 ± 11.15		DT56–60 Gy	esophageal cancer,				
				(1.8–2.0 Gy/f)	breast cancer				
Zhang^[36]^	45/45	57.5 ± 9.4	51/39	RT	lung cancer,	-	Huangqisanshen Yin	per os	①③④
(2007)		55.6 ± 8.2		DT ≥ 8 Gy	esophageal cancer,				
				(2 Gy/f)	breast cancer				
Xia^[37]^	30/30	50 ± 10	27/33	CRT + 3D-CRT	lung cancer,	-	Qingzaojiufei Tang and Huangqijing Oral Liquid	per os	②④
(2010)		50 ± 10		DT30–75 Gy	esophageal cancer,				
					breast cancer				
Xie^[38]^	46/51	-	68/29	3D-CRT	NSCLC	IIIa,	Shenqifuzheng injection	intravenous drip	①
(2009)				DT60–70 Gy		IIIb,			
						IV			
Dong^[39]^	30/30	56.95 ± 13.30	43/17	RT	NSCLC	I,	Yangyinshengjin Fang	per os	①③⑤
(2007)		57.25 ± 11.2				II,			
						III,			
						IV			
Jin^[40]^	24/22	58.65 ± 13.69	35/11	RT	NSCLC	-	Yiqiyangyin Fang	per os	①③
(2005)		59.86 ± 11.23							
Ji^[41]^	30/30	58.2 ± 6.4	36/24	RT	NSCLC	I,	Shenqigujin Tang	per os	①④
(2017)		57.1 ± 7.0				II,			
						III,			
						IV			
Zheng^[42]^	48/32	61.3 ± 0.2	53/27	CRT	adenocarcinoma,	-	Shenqifuzheng injection	intravenous drip	①⑤
(2007)		60.5 ± 0.3		DT55–60 Gy	squamous cell carcinoma				
Ni^[43]^	51/50	59.2	56/45	IMRT	NSCLC	IIIa,	Shuizhihuangqi	per os	②
(2021)		59.5				IIIb,	Tang		
Gong^[44]^	16/16	Total	22/10	CRT	lung cancer,		Xianyu Tang	per os	②④
(2012)		18~75		DT40–70 Gy	thymus cancer,				
				(1.8–2.0 Gy/f)	esophageal cancer,				
					breast cancer				

- = no data or no special instructions, ① = recent response evaluation (RECIST), ② = acute radiation injury, ③ = Cancer patient quality of life score (KPS), ④ = Cytokine levels, ⑤ = TCM clinical symptoms improvement or integral change, 3DCRT = three-dimensional conformal radiotherapy, C = control group, CRT = 6MV-X-ray, linear accelerator irradiation, F = female, IMRT = intensity modulated radiation therapy, M = male, NSCLC = non-small cell lung cancer, T = treatment group.

### 3.2. Risk of bias assessment of included studies

**Figure 2.**
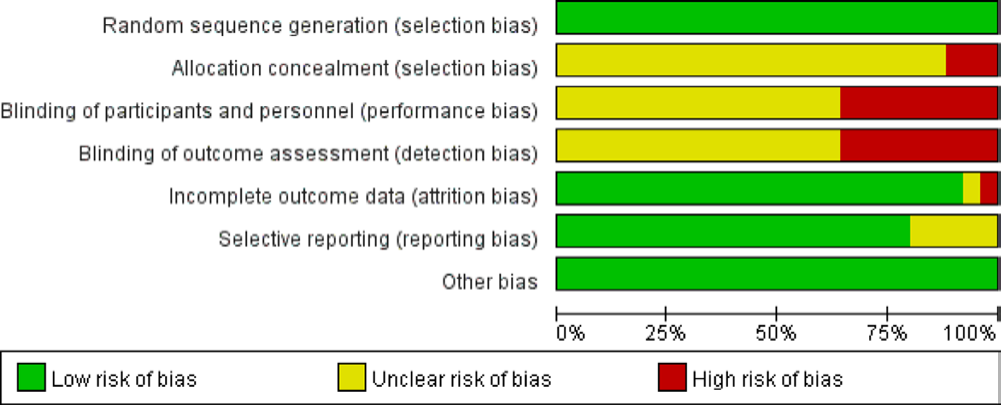
Risk of bias graph.

**Figure 3.**
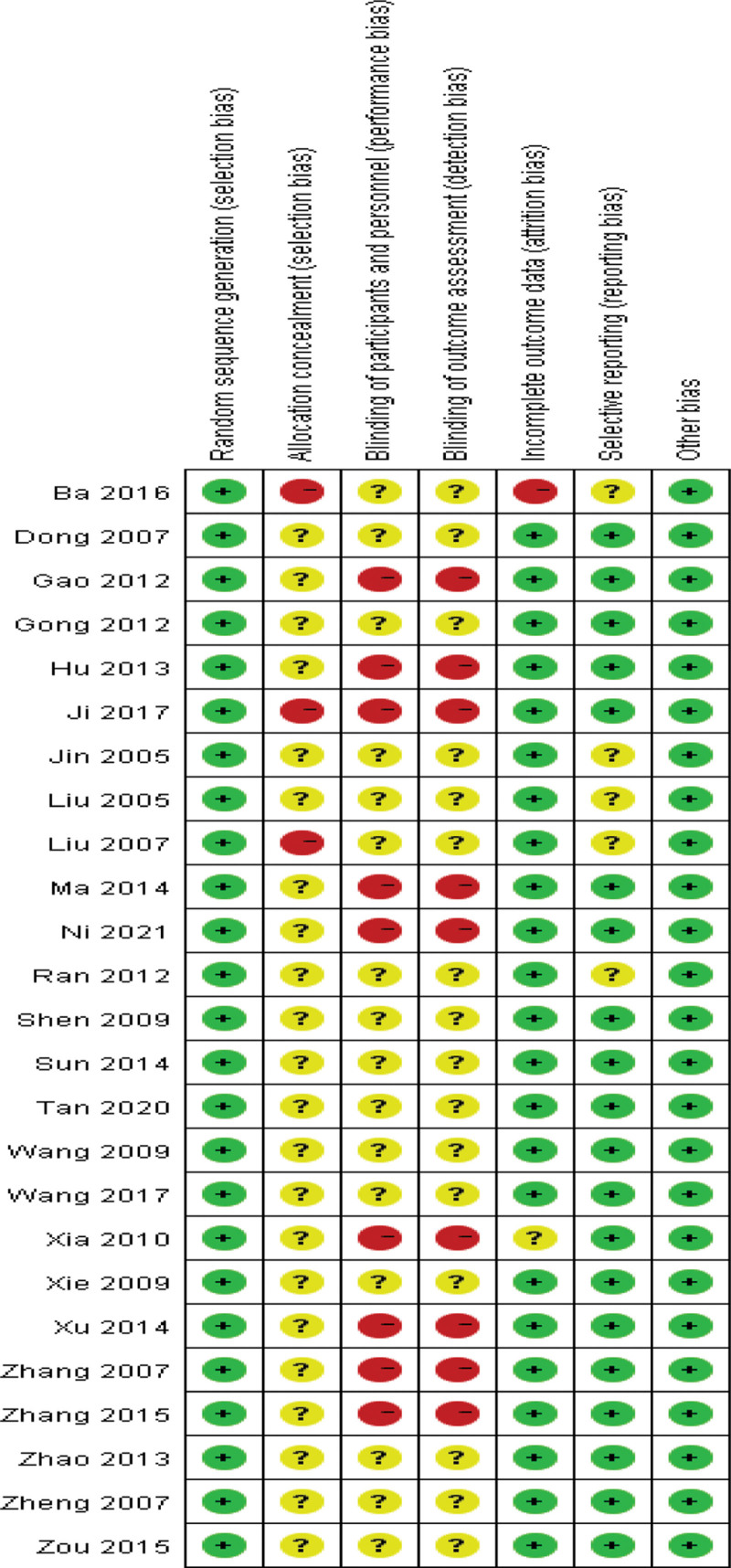
Risk of bias summary.

Risk of bias for each study was assessed according to section 5.1.0 of the Cochrane Handbook for Systematic Reviews of Interventions. Twenty-five studies were randomized, and 3 of them reported using a random number table method and were therefore assessed as “low risk.” One study used randomization of patients and one study used randomization coding and was assessed as “high risk.” Other studies did not report any randomization procedures, the allocation scheme was concealed and was assessed as “unclear.” In 9 studies, patients or their family members signed the informed consent form and were assessed as “unblinded” and assessed as “high risk,” while the remaining studies had difficulty in blinding the research results, and the design blinding method was assessed as “unclear.” One outcome data were incomplete, and the sample sizes of included studies and outcome data were inconsistent and were assessed as “high risk,” and studies that did not report missing data were assessed as “unclear.” Five studies with study selection bias were assessed as “unclear,” and the remaining studies were assessed as “low risk” due to insufficient information to assess selection bias in these studies. The general clinical data of all studies were not statistically different, the baseline was balanced, and it was assessed as “low risk.” Its literature quality evaluation chart is shown in Figures 2 and 3.

### 3.3. Recent response evaluation

#### 3.3.1. Heterogeneity test.

For the 15 literatures in this study, after the heterogeneity test, *I*^2^ = 0% < 50%, *P* = .90 > .1 of the *Q* test, indicating that the heterogeneity of the literatures selected in this study is not statistically significant, the fixed-effects model is selected to determine the effect size.

#### 3.3.2. Fixed effects meta-analysis.

**Figure 4.**
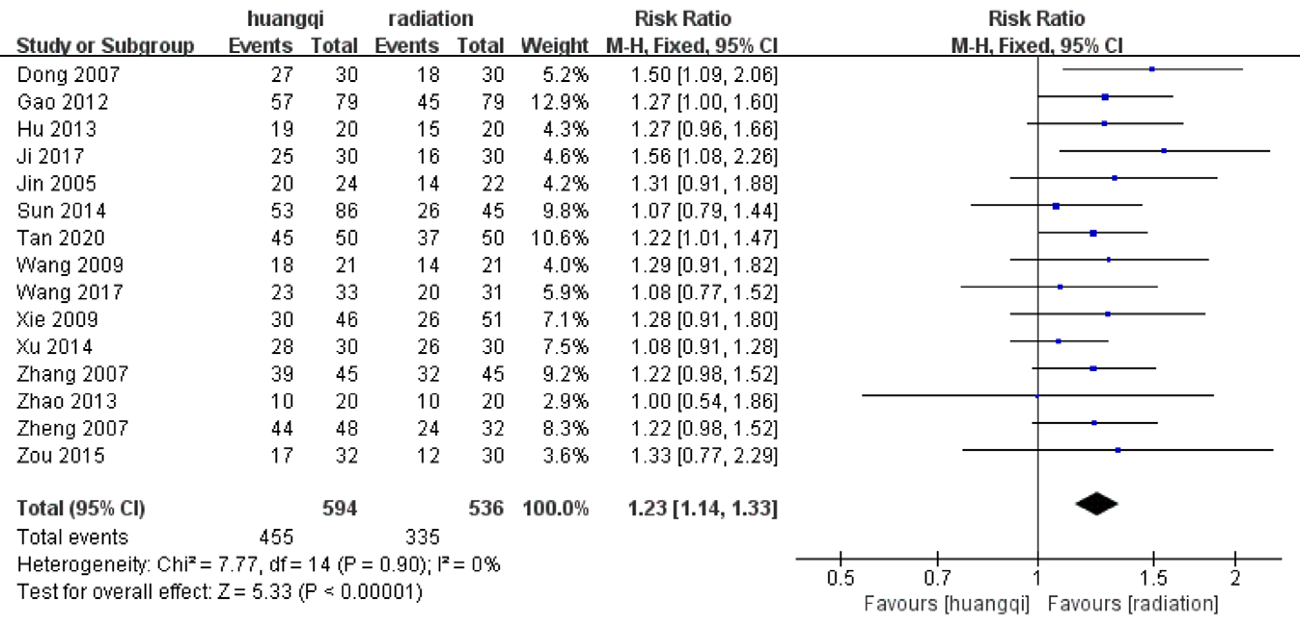
Forest plot fixed effects analysis of therapeutic effect (Huangqi is equivalent to traditional Chinese medicine preparations containing astragalus; Radiation is equivalent to radiation therapy).

The combined effect size of the fixed models of the 15 studies was RR = 1.23 (1.14–1.33), which was statistically significant, *Z* = 5.33, *P* < .00001, indicating that the efficacy of traditional Chinese medicinal preparations containing astragalus in the treatment of RILI was significantly better than that of the RT group, the efficacy of traditional Chinese medicinal preparations containing astragalus was 1.23 times that of the RT group (*P* < .05), as shown in the following (Fig. 4).

#### 3.3.3. Bias test.

**Figure 5.**
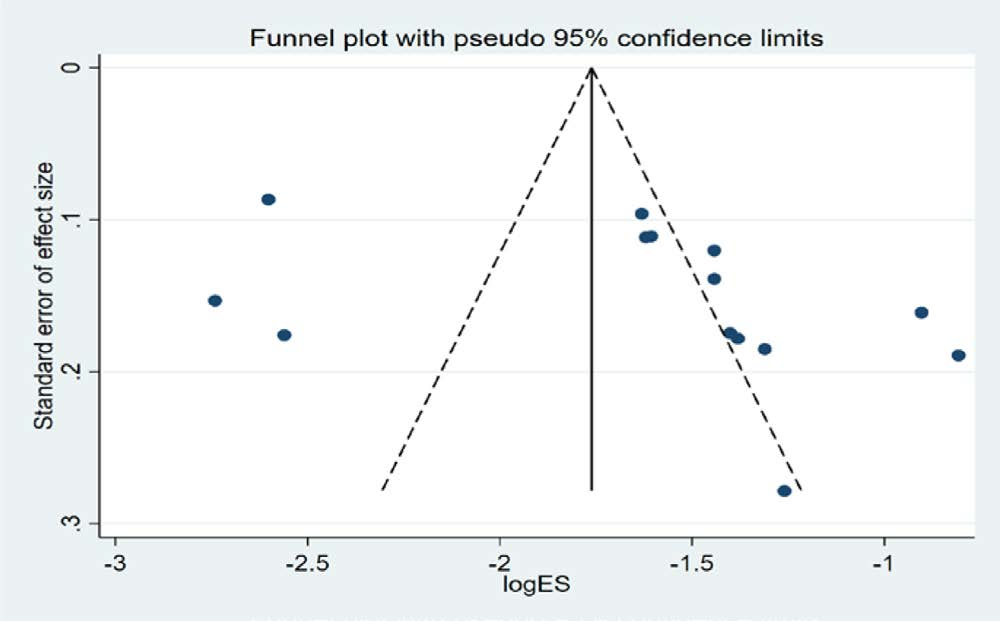
Funnel plot bias test of therapeutic effect.

The funnel plot was drawn by STATA to investigate whether there is publication bias in the fifteen papers in this study. The funnel plot obtained is symmetrical (Egger test *P* = .137 > .05), indicating that there is no publication bias, and the conclusion of this study is accurate and reliable, as shown in the following (Fig. 5).

### 3.4. Acute radiation injury

#### 3.4.1. Heterogeneity test.

For the 10 literatures in this study, after the heterogeneity test, *I*^2^ = 0% < 50%, *P* = .94 > .1 of the *Q* test, indicating that the heterogeneity of the literatures selected in this study is not statistically significant, the fixed-effects model is selected to determine the effect size.

#### 3.4.2. Fixed effects meta-analysis.

**Figure 6.**
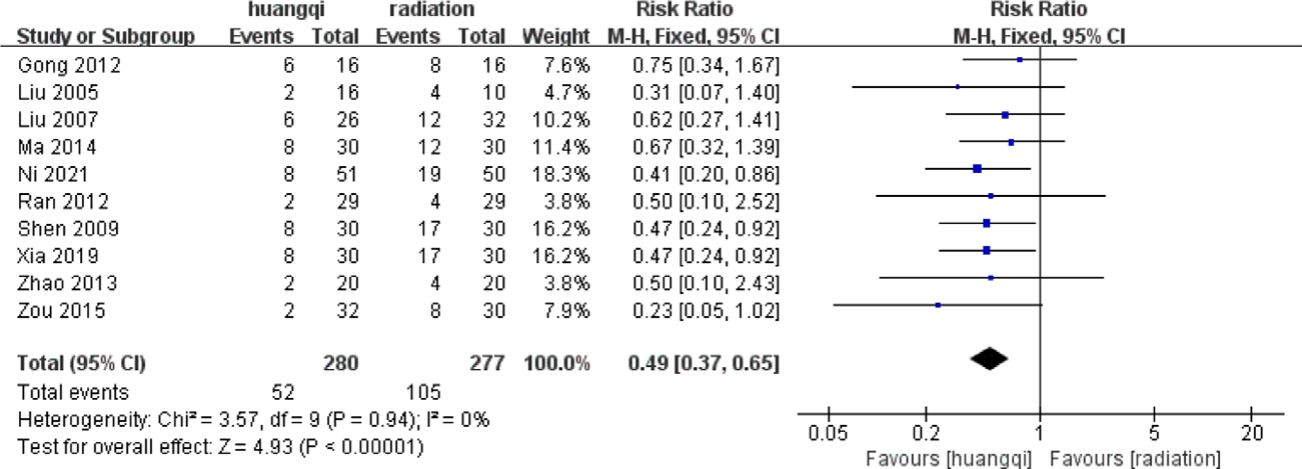
Forest plot fixed effects analysis of incidence of radiation-induced lung injury. Notes: Huangqi is equivalent to traditional Chinese medicine preparations containing astragalus; Radiation is equivalent to radiation therapy.

The combined effect size of the fixed models of the 10 studies was RR = 0.49 (0.37–0.65), which was statistically significant, *Z* = 4.93, *P* < .00001, indicating that the incidence of adverse events in the treatment of RILI with traditional Chinese medicinal preparations containing astragalus is significantly lower than that in the RT group alone, the incidence of RILI in traditional Chinese medicinal preparations containing astragalus was 0.49 times that of the RT group (*P* < .05), as shown in the following (Fig. 6).

#### 3.4.3. Bias test.

**Figure 7.**
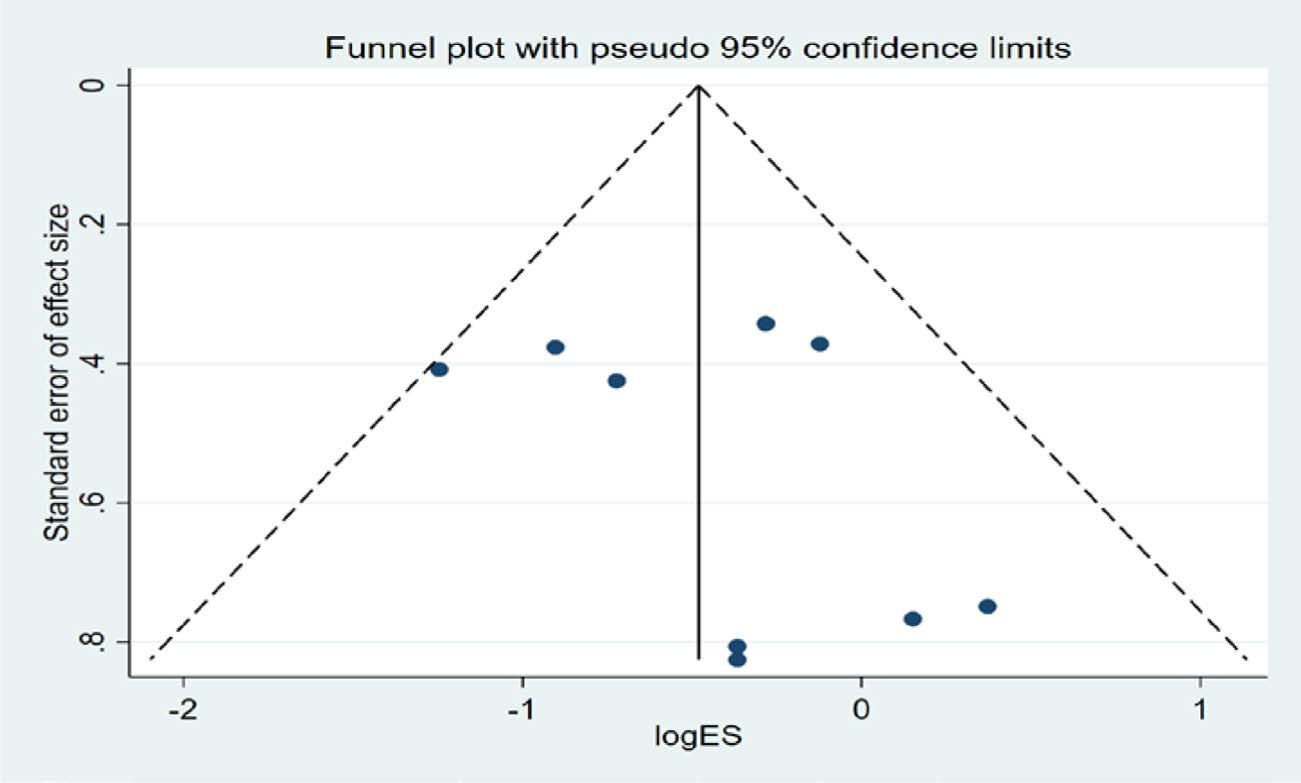
Funnel plot bias test of incidence of radiation-induced lung injury.

The funnel plot was drawn by STATA to investigate whether there is publication bias in the ten papers in this study. The funnel plot obtained is symmetrical (Egger test *P* = .450 > .05), indicating that there is no publication bias, and the conclusion of this study is accurate and reliable, as shown in the following (Fig. 7).

### 3.5. Cancer patient quality of life score

#### 3.5.1. Heterogeneity test.

For the 5 literatures in this study, after the heterogeneity test, *I*^2^ = 1% < 50%, *P* = .40 > .1 of the *Q* test, indicating that the heterogeneity of the literatures selected in this study is not statistically significant, the fixed-effects model is selected to determine the effect size.

#### 3.5.2. Fixed effects meta-analysis.

**Figure 8.**
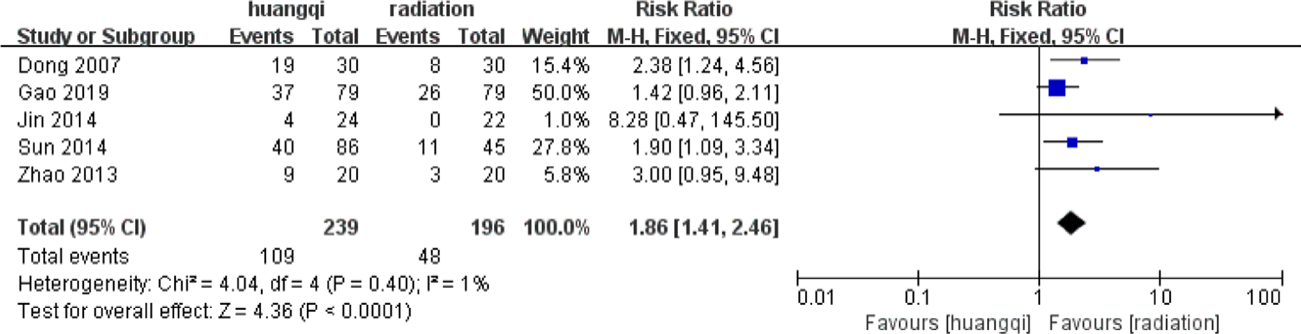
Forest plot fixed effects analysis of the KPS score. Notes: Huangqi is equivalent to traditional Chinese medicine preparations containing astragalus; Radiation is equivalent to radiation therapy. KPS = cancer patient quality of life score.

The combined effect size of the fixed models of the 10 studies was RR = 1.86 (1.41–2.46), which was statistically significant, *Z* = 4.36, *P* < .00001, indicating that the KPS score of traditional Chinese medicinal preparations containing astragalus in the treatment of RILI was significantly higher than that of the RT group, which could effectively improve the quality of life of patients, and the incidence of RILI in the traditional Chinese medicinal preparations containing astragalus was 1.86 times that of the RT group (*P* < .05), as shown in the following (Fig. 8).

#### 3.5.3. Bias test.

**Figure 9.**
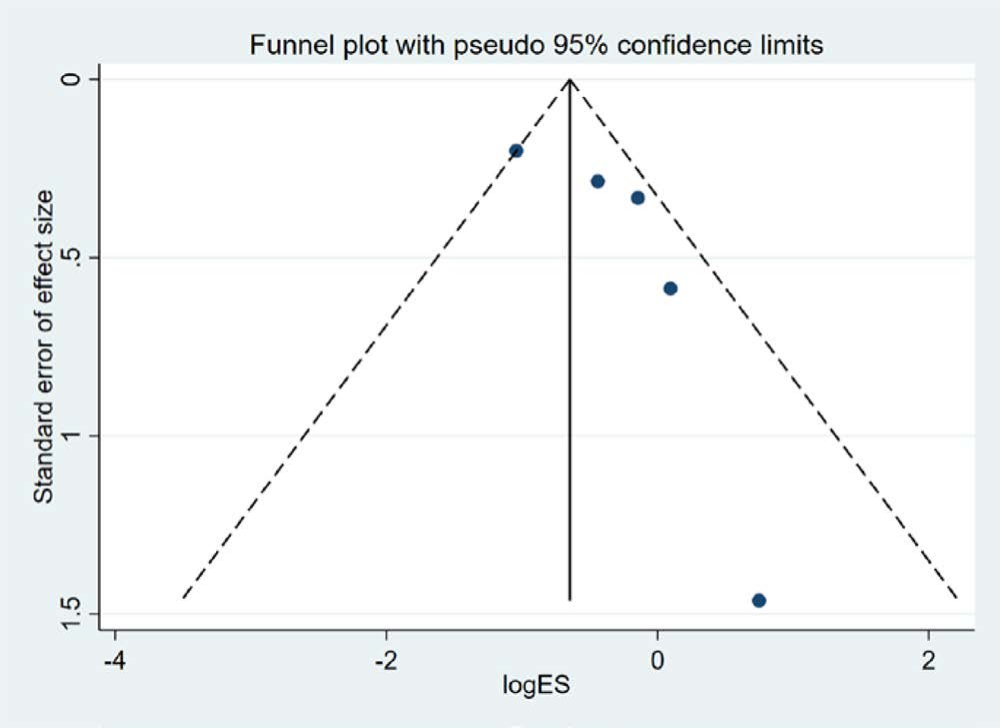
Funnel plot bias test of the KPS score. KPS = cancer patient quality of life score.

The funnel plot was drawn by STATA to investigate whether there is publication bias in the 5 papers in this study. The funnel plot obtained is symmetrical (Egger test *P* = .125 > .05), indicating that there is no publication bias, and the conclusion of this study is accurate and reliable, as shown in the following (Fig. 9).

### 3.6. Serum cytokines

#### 3.6.1. Baseline consistency test.

**Figure 10.**
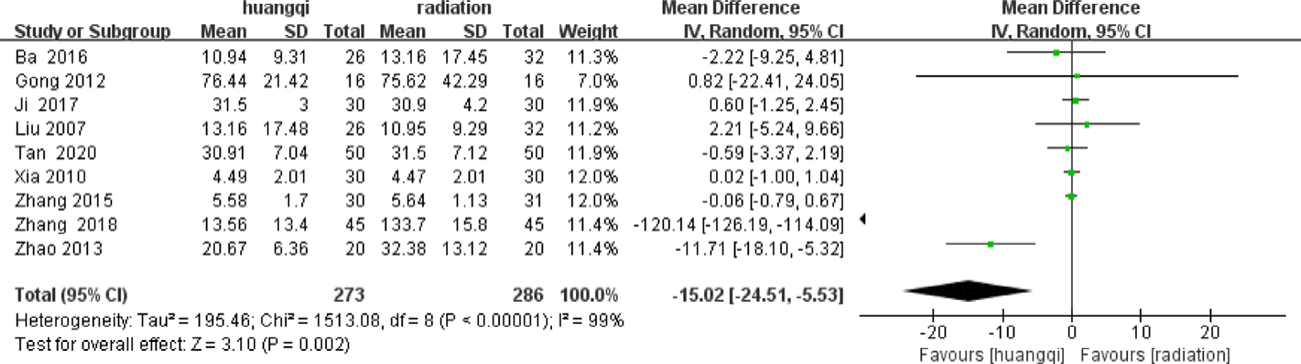
Forest plot baseline consistency test of TGF-β levels. Notes: Huangqi is equivalent to traditional Chinese medicine preparations containing astragalus; Radiation is equivalent to radiation therapy. TGF-β = transforming growth factor (TGF)-β.

Before performing meta-analysis, it is necessary to ensure that the baseline periods of the 2 groups of data are consistent before subsequent meta-analysis can be performed, as shown in the following (Fig. 10).

It can be clearly seen from the above forest plot that the baseline difference of TGF-β in the baseline period of the 2 groups of data is *I*^2^ = 99% > 50%, *P* < .00001, there is heterogeneity, and a random effect model is used to combine TGF-β the difference between the 2 groups in the baseline period, TGF-β levels in patients is (standard mean difference = −15.022, 95% confidence interval [−24.512, −5.5320], *P* = .002).

#### 3.6.2. Heterogeneity test.

**Figure 11.**
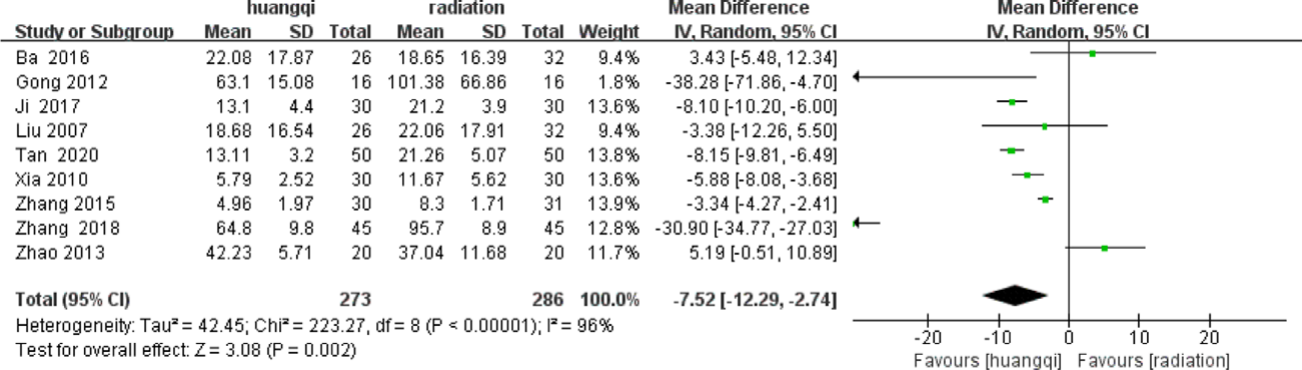
Forest plot heterogeneity test of TGF-β levels. Notes: Huangqi is equivalent to traditional Chinese medicine preparations containing astragalus; Radiation is equivalent to radiation therapy. TGF-β = transforming growth factor (TGF)-β.

After the heterogeneity test of the 9 literatures in this study, *I*^2^ = 96% > 50%, and the *Q* test, *P* < .1, indicating that the literatures selected in this study have strong heterogeneity. The random effect model was used to show that the improvement of serum TGF-β in patients with RILI was not statistically significant with traditional Chinese medicinal preparations containing astragalus, as shown in the following (Fig. 11).

### 3.7. Clinical symptoms of TCM

#### 3.7.1. Chest tightness chest pain.

##### 3.7.1.1. Heterogeneity test.

For the 3 literatures in this study, after the heterogeneity test, *I*^2^ = 0% < 50%, *P* = .45 > .1 of the *Q* test, indicating that the heterogeneity of the literatures selected in this study is not statistically significant, the fixed-effects model is selected to determine the effect size.

##### 3.7.1.2. Fixed effects meta-analysis.

**Figure 12.**
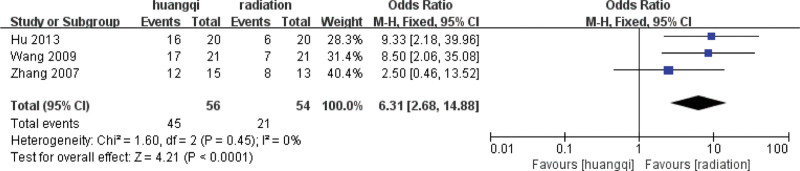
Forest plot fixed effects analysis of the improvement of chest tightness and chest pain symptoms. Notes: Huangqi is equivalent to traditional Chinese medicine preparations containing astragalus; Radiation is equivalent to radiation therapy.

The combined effect size of the fixed model of the 3 studies was RR = 6.31 (2.68, 14.88), which was statistically significant, *Z* = 4.21, *P* < .00001, indicating that the traditional Chinese medicinal preparations containing astragalus can effectively relieve the symptoms of chest tightness and chest pain mixed with RILI. The efficacy of traditional Chinese medicinal preparations containing astragalus was 6.31 times that of the RT group (*P* < .05), as shown in the following (Fig. 12).

##### 3.7.1.3. Bias test.

**Figure 13.**
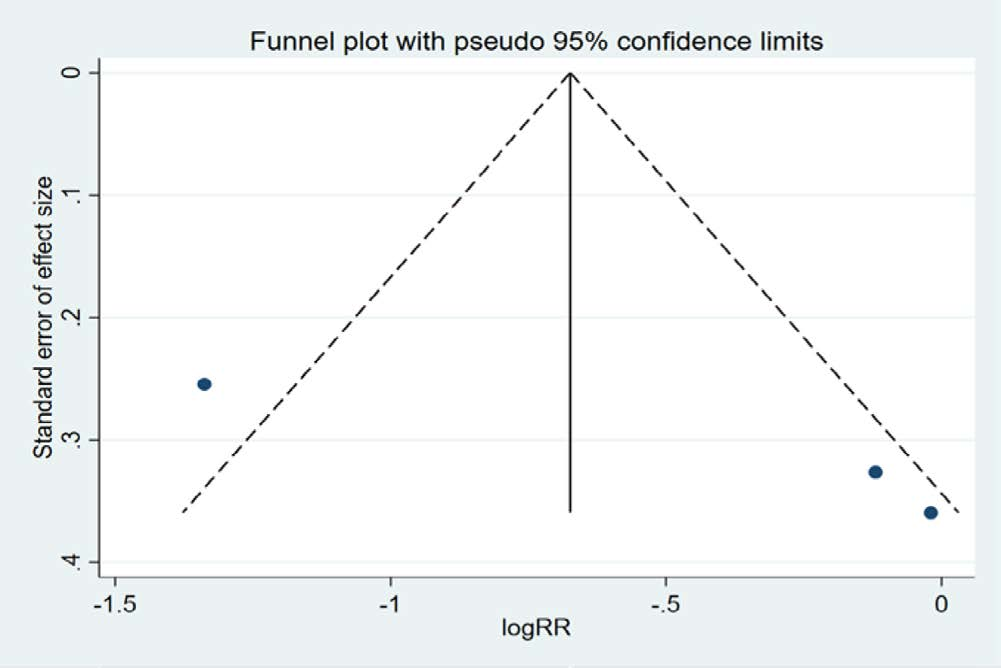
Funnel plot bias test of the improvement of chest tightness and chest pain symptoms.

The funnel plot was drawn by STATA to investigate whether there is publication bias in the 3 papers in this study. The funnel plot obtained is symmetrical (Egger test *P* = .135 > .05), indicating that there is no publication bias, and the conclusion of this study is accurate and reliable, as shown in the following (Fig. 13).

#### 3.7.2. Cough and hemoptysis.

##### 3.7.2.1. Heterogeneity test.

**Figure 14.**
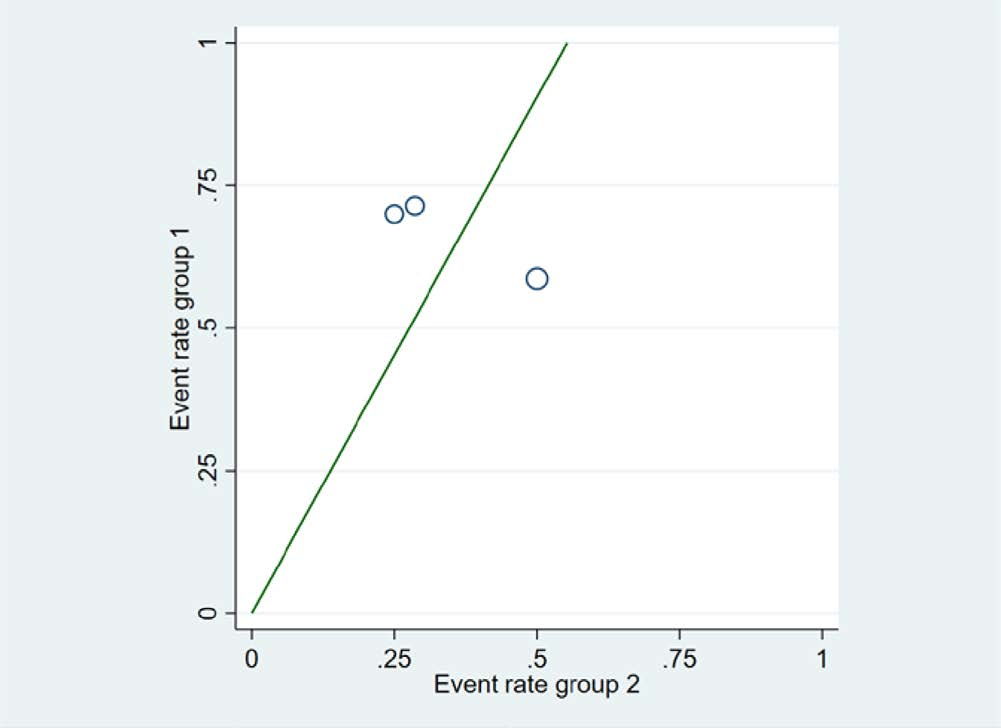
Rabet plot heterogeneity test of the improvement of shortness of cough and hemoptysis symptoms.

**Figure 15.**
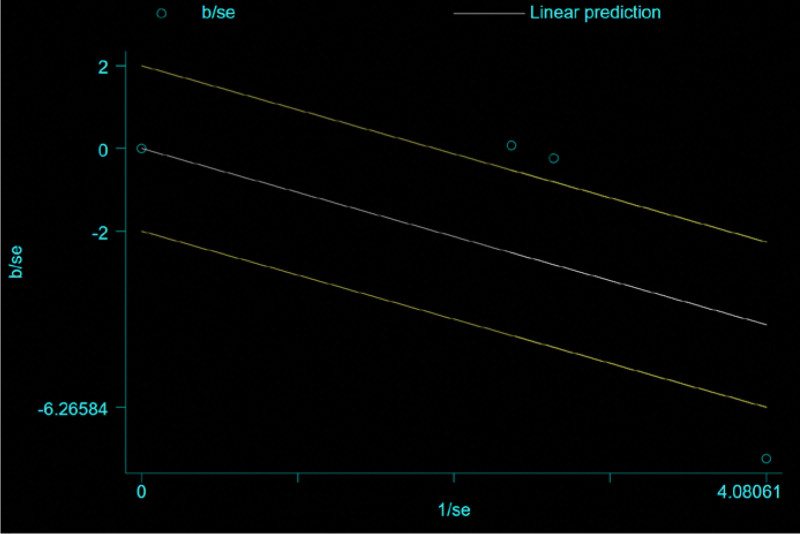
Galbr plot heterogeneity test of the improvement of cough and hemoptysis symptoms.

For the 3 literatures in this study, after the heterogeneity test, *I*^2^ = 60% > 50% of the *Q* test, *P* = .08 < .1, indicating that the literature selected in this study has heterogeneity, there is difference in the data between the 2 groups. Statistical significance requires further investigation of the rabet map and star map, suggesting that there is a literature with strong heterogeneity, as shown in the following (Figs. 14 and 15).

##### 3.7.2.2. Random effects meta-analysis.

**Figure 16.**
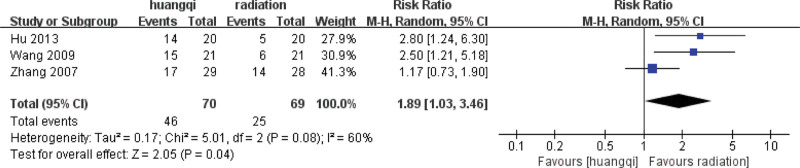
Forest plot random effects analysis of the improvement of cough and hemoptysis symptoms. Notes: Huangqi is equivalent to traditional Chinese medicine preparations containing astragalus; Radiation is equivalent to radiation therapy.

The combined effect size of the random model of the 3 studies was RR = 1.89 (1.03, 3.46), *Z* = 2.54, *P* = .04 < .05, which was statistically significant, indicating that the traditional Chinese medicinal preparations containing astragalus can effectively relieve cough and hemoptysis symptoms compared with RILI. The symptoms of cough and hemoptysis symptoms and the efficacy of the traditional Chinese medicinal preparations containing astragalus was 1.89 times that of the RT group (*P* < .05), as shown in the following (Fig. 16).

##### 3.7.2.3. Sensitivity analysis.

**Figure 17.**
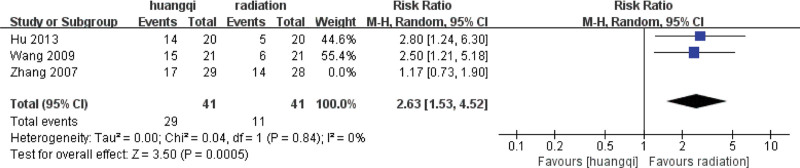
Forest plot sensitivity analysis of cough and hemoptysis symptoms. Notes: Huangqi is equivalent to traditional Chinese medicine preparations containing astragalus; Radiation is equivalent to radiation therapy.

Sensitivity analysis was performed on the selected 3 literatures. After excluding the study of Zhang (2007), it was found that *I*^2^ = 0%, *P* = .84 > .1, and there was no heterogeneity in the 2 groups of data, indicating that the difference in intervention measures caused the existence of heterogeneity. For heterogeneity reasons, 1 group is for nourishing lung qi, and the other group is for heat-clearing and blood-activating, as shown in the following (Fig. 17).

##### 3.7.2.4. Subgroup based analysis.

The heterogeneity of the benifit qi group (*I*^2^ = 0%, *P* = .84 > .1) was not statistically significant, then the fixed effect model was selected to combine the effect size, and the RR = 2.63 (1.53, 4.52) was obtained, indicating that traditional Chinese medicinal preparations containing astragalus (TCM for benifiting qi) improved the symptoms of cough and hemoptysis symptoms by traditional Chinese medicinal preparations containing astragalus was 2.63 times that of the RT group, which was statistically significant (*Z* = 3.50, *P* = .0005 < .05).

**Figure 18.**
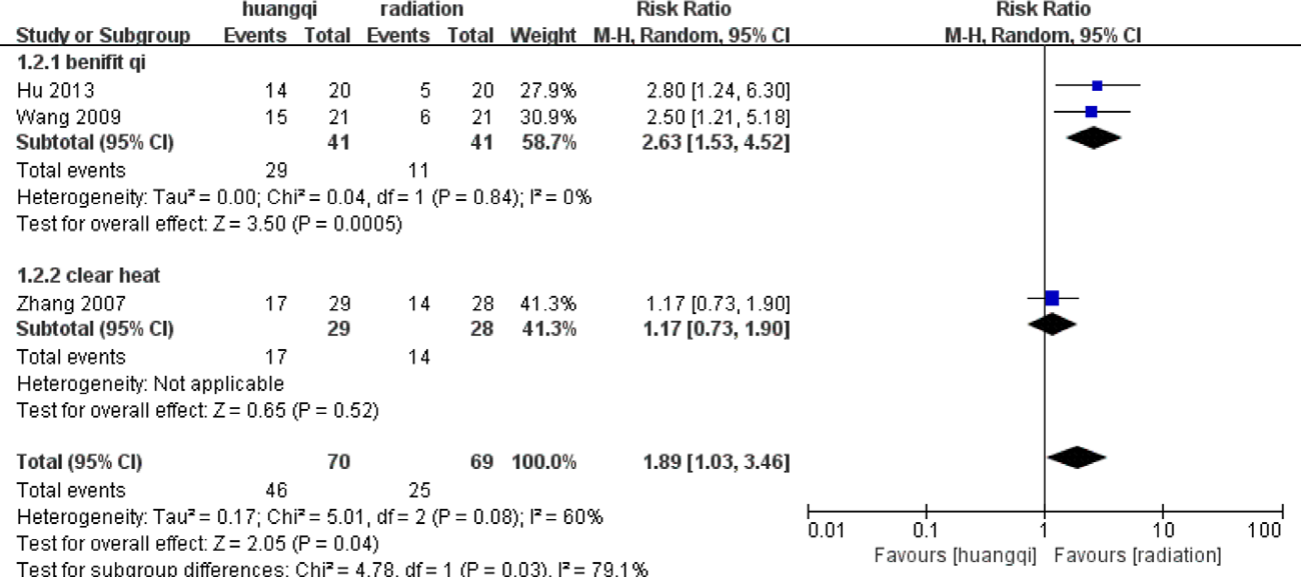
Forest plot subgroup analysis cough and hemoptysis symptoms. Notes: Huangqi is equivalent to traditional Chinese medicine preparations containing astragalus; Radiation is equivalent to radiation therapy.

There is only one study in the heat-clearing group, and there is no *I*^2^ and *P* value. The fixed-effect model was selected to combine the effect size, and the RR = 1.17 (0.73, 1.90) was obtained, indicating that the traditional Chinese medicinal preparations containing astragalus (TCM for clearing heat) improved the symptoms of cough and hemoptysis 1.17 times, and the difference was not statistically significant (*Z* = 0.65, *P* = .52 > .05), as shown in the following (Fig. 18).

##### 3.7.2.5. Bias test.

**Figure 19.**
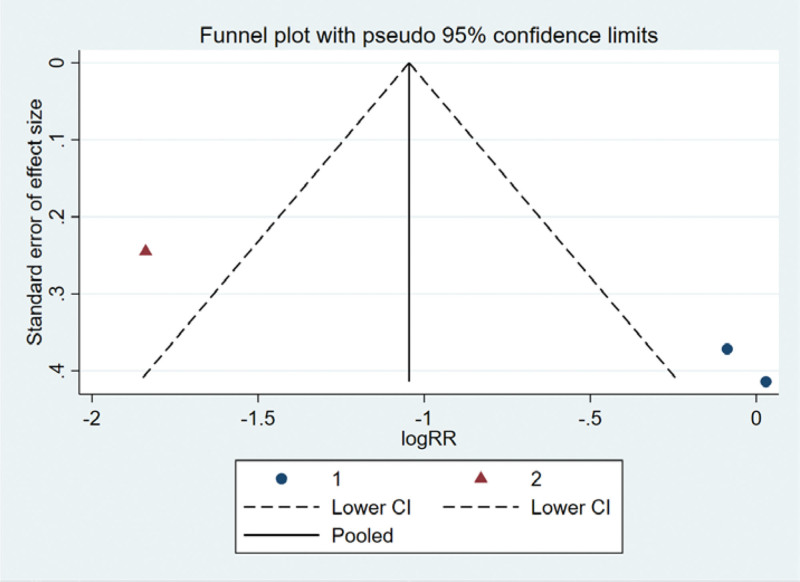
Funnel plot bias test of improvement of cough and hemoptysis symptoms.

The funnel plot was drawn by STATA to investigate whether there is publication bias in the 3 papers in this study. The funnel plot obtained is symmetrical (Egger test *P* = .094 > .05), indicating that there is no publication bias, and the conclusion of this study is accurate and reliable, as shown in the following (Fig. 19).

#### 3.7.3. Shortness of breath.

##### 3.7.3.1. Heterogeneity test.

**Figure 20.**
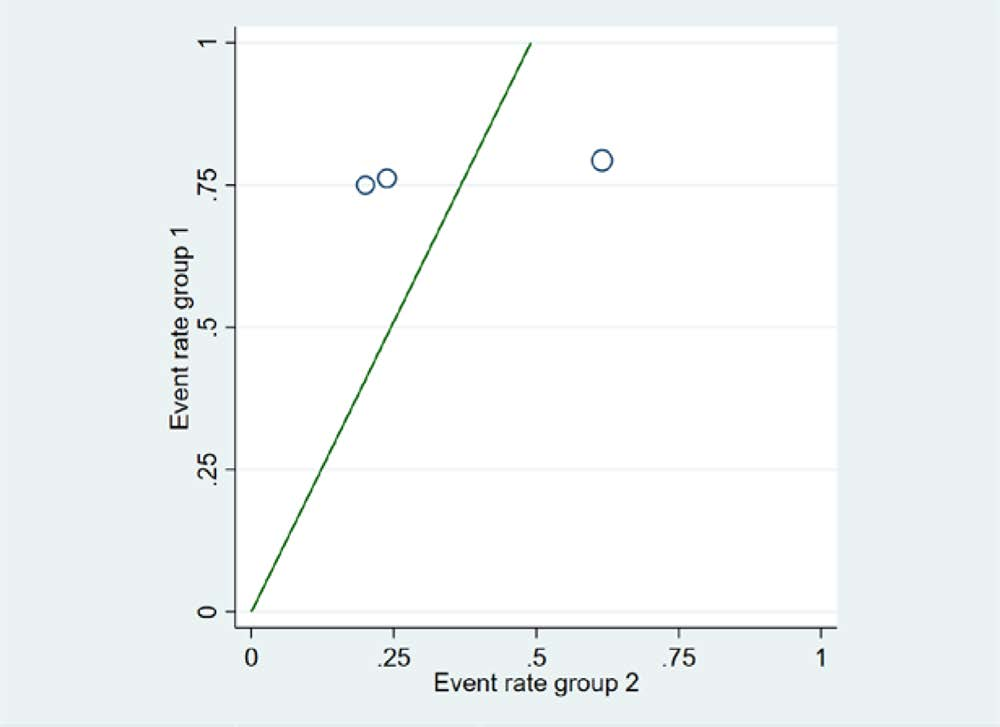
Rabet plot heterogeneity test of the improvement of shortness of breath symptoms.

**Figure 21.**
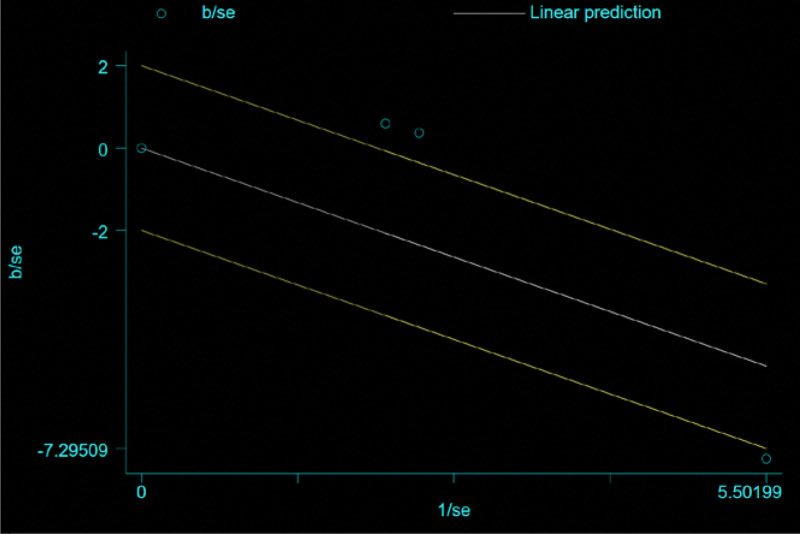
Galbr plot heterogeneity test of the improvement of shortness of breath symptoms.

For the 3 literatures in this study, after the heterogeneity test, *I*^2^ = 78% > 50% of the *Q* test, *P* = .1, indicating that the literature selected in this study has heterogeneity, there is difference in the data between the 2 groups. Statistical significance requires further investigation of the rabet map and star map, suggesting that there is a literature with strong heterogeneity, as shown in the following (Figs. 20 and 21).

##### 3.7.3.2. Random effects meta-analysis.

**Figure 22.**
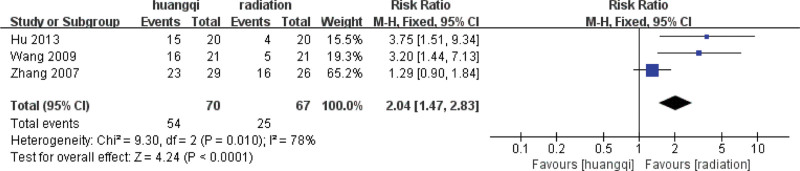
Forest plot random effects analysis of the improvement of shortness of breath symptoms. Notes: Huangqi is equivalent to traditional Chinese medicine preparations containing astragalus; Radiation is equivalent to radiation therapy.

The combined effect size of the random model of the 3 studies was RR = 2.04 (1.47, 2.83), *Z* = 4.24, *P* < .0001, which was statistically significant, indicating that the traditional Chinese medicinal preparations containing astragalus can effectively relieve shortness of breath compared with RILI. The symptoms of shortness of breath and the efficacy of the traditional Chinese medicinal preparations containing astragalus was 1.89 times that of the RT group (*P* < .05), as shown in the following (Fig. 22).

##### 3.7.3.3. Sensitivity analysis.

**Figure 23.**
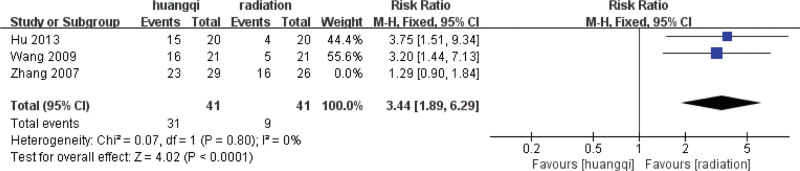
Forest plot sensitivity analysis of the improvement of shortness of breath symptoms. Notes: Huangqi is equivalent to traditional Chinese medicine preparations containing astragalus; Radiation is equivalent to radiation therapy.

Sensitivity analysis was performed on the selected 3 literatures. After excluding the study of Zhang (2007), it was found that *I*^2^ = 0%, *P* = .80 > .1, and there was no heterogeneity in the 2 groups of data, indicating that the difference in intervention measures caused the existence of heterogeneity. For heterogeneity reasons, 1 group is for nourishing lung qi, and the other group is for heat-clearing and blood-activating, as shown in the following (Fig. 23).

##### 3.7.3.4. Subgroup based analysis.

The heterogeneity of the qi-benefiting group (*I*^2^ = 0%, *P* = .80 > .1) was not statistically significant, then the fixed effect model was selected to combine the effect size, and the RR = 3.43 (1.88, 6.26) was obtained, indicating that traditional Chinese medicinal preparations containing astragalus (TCM for benefiting qi) improved the symptoms of shortness of breath symptoms by traditional Chinese medicinal preparations containing astragalus was 3.43 times that of the RT group, which was statistically significant (*Z* = 4.01, *P* < .0001).

**Figure 24.**
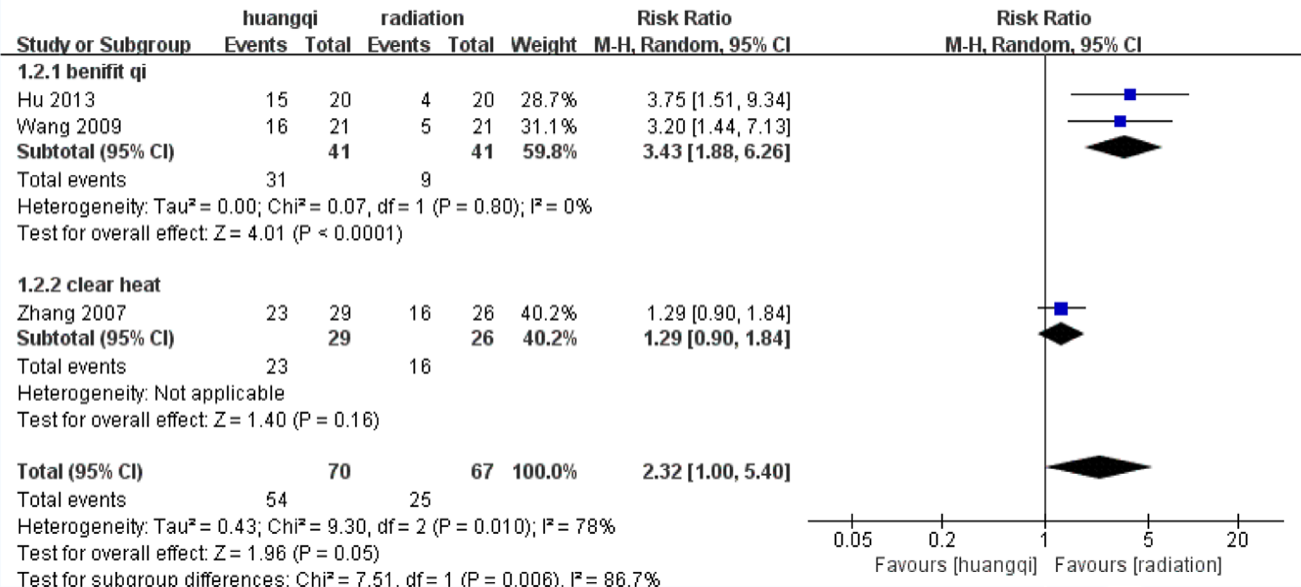
Forest plot subgroup analysis of the improvement of shortness of breath symptoms. Notes: Huangqi is equivalent to traditional Chinese medicine preparations containing astragalus; Radiation is equivalent to radiation therapy.

There is only 1 study in the heat-clearing group, and there is no *I*^2^ and *P* value. The fixed-effect model was selected to combine the effect size, and the RR = 1.29 (0.90, 184) was obtained, indicating that traditional Chinese medicinal preparations containing astragalus (TCM for clearing heat) improved the symptoms of shortness of breath 1.29 times, and the difference was not statistically significant (*Z* = 1.40, *P* = .16 > .05), as shown in the following (Fig. 24).

##### 3.7.3.5. Bias test.

**Figure 25.**
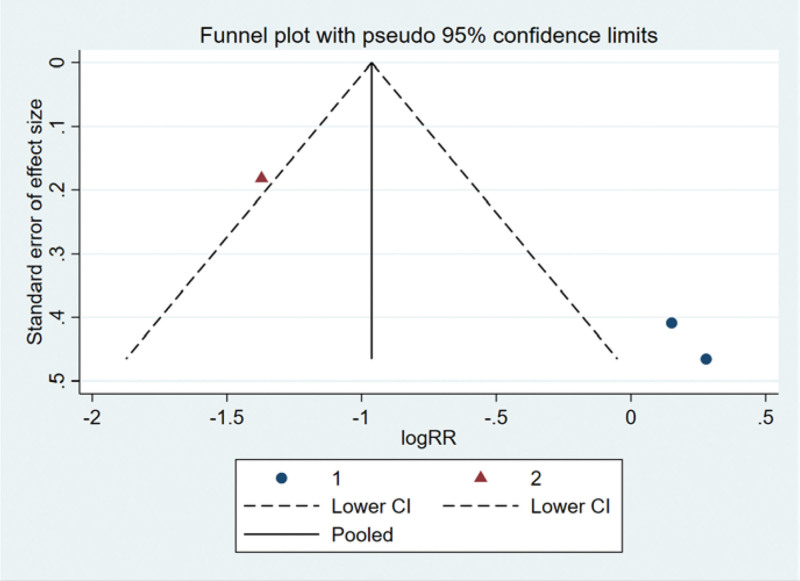
Funnel plot bias analysis of the improvement of shortness of breath symptoms.

The funnel plot was drawn by STATA to investigate whether there is publication bias in the 3 papers in this study. The funnel plot obtained is symmetrical (Egger test *P* = .052 > .05), indicating that there is no publication bias, and the conclusion of this study is accurate and reliable, as shown in the following (Fig. 25).

## 4. Discussion

Radiation therapy is one of the treatments for a variety of malignant tumors, including lung cancer, breast cancer, esophageal cancer, prostate cancer, branch rectal cancer, etc. With the advancement of RT technology, such as 3D conformal radiotherapy, intensity modulated RT, image guided radiotherap, stereotactic body radiotherapy,^[45]^ cancer cells can be effectively and precisely killed, but radiation dose is still an important factor limiting RT and can cause radiation damage. RILI can be divided into early acute RP and chronic RPF.^[46]^ The pathogenesis of RILI is a very complex histological and physiological process, mainly including direct DNA damage and repair, as well as the generation of free reactive oxygen species, activation of the renin-angiotensin-aldosterone system, epithelial-mesenchymal transition activation.^[47]^ The current treatment of RILI is mainly to improve the symptoms of chest tightness, chest pain, dry cough and hemoptysis, and shortness of breath, and to take corresponding anti-inflammatory treatment and corticosteroid treatment.^[48]^ The effect of astragalus is to nourish qi and strengthen the lungs and spleen. Astragalus contains active compounds such as astragalus saponins and astragalus polysaccharides, which have potential roles in boosting the immune system, in the treatment of inflammation, and in the treatment of many types of cancer.^[49,50]^

This study mainly studied 25 literatures (n = 1762) using traditional Chinese medicinal preparations containing astragalus including decoction, injection and other formulations, and observed and studied the clinical efficacy evaluation, quality of life (KPS score), the symptom scores and cellular inflammatory factors.

The recent efficacy evaluation of 15 studies showed that the traditional Chinese medicinal preparations containing astragalus can effectively improve the efficacy of RILI compared with the RT group; 10 studies showed that the use of traditional Chinese medicinal preparations containing astragalus can reduce RILI; 10 studies showed that the KPS score of traditional Chinese medicinal preparations containing astragalus in the treatment of RILI was significantly higher than that of the RT group, which could effectively improve the quality of life of patients; 3 literatures showed that traditional Chinese medicinal preparations containing astragalus could improve the clinical symptoms of patients with RILI include chest tightness and chest pain, dry cough and hemoptysis, and shortness of breath. At the same time, the improvement of the serum cytokine TGF-β in this study was not statistically significant, and further clinical research and investigation are needed.

At the same time, this meta-analysis has certain limitations: only 25 literatures were included, and most of the literatures were single-center, small-sample trials; all indicators of RILI assessment were not involved; the background, location, and specific methods of each study were included. Inconsistent, there may be some confounding bias in the unified meta-analysis. In clinical application, due to differences in radiation techniques and radiation doses; inconsistencies in interventions such as TCM formulations; and inconsistencies in long-term efficacy such as readmission rates and regular follow-up; the trial results may be biased. We expect more high-quality, large-sample, multi-center RCTs to further validate the results of this study.

## 5. Conclusion

Overall, this systematic review and meta-analysis suggests that traditional Chinese medicinal preparations containing astragalus can alleviate clinical symptoms, improve short-term efficacy, and improve patients’ quality of life in patients with RILI. However, higher quality RCTs are still needed to further demonstrate their efficacy and provide more meaningful evidence.

## Author contributions

**Conceptualization:** Xue-Meng Pang, Xin Zheng.

**Data curation:** Jing-Jing Shi, Jie Zhao.

**Formal analysis:** Ping-Yi Sun.

**Investigation:** Juan Liu, Zong-Chen Liu.

**Methodology:** Xue-Meng Pang, Xin Zheng.

**Software:** Xue-Meng Pang, Yan-Li Zhang.

**Supervision:** Hou-Hao Cai.

**Visualization:** Xue-Meng Pang.

**Writing – original draft:** Xue-Meng Pang.
